# Sequential determination of serum viral titers, virus-specific IgG antibodies, and TNF-α, IL-6, IL-10, and IFN-γ levels in patients with Crimean-Congo hemorrhagic fever

**DOI:** 10.1186/1471-2334-14-416

**Published:** 2014-07-28

**Authors:** Safak Kaya, Nazif Elaldi, Ayhan Kubar, Nevcihan Gursoy, Meral Yilmaz, Gulderen Karakus, Turabi Gunes, Zubeyde Polat, Mustafa Gokhan Gozel, Aynur Engin, Ilyas Dokmetas, Mehmet Bakir, Neziha Yilmaz, Mehmet Sencan

**Affiliations:** Department of Infectious Diseases and Clinical Bacteriology, Training and Research Hospital, Diyarbakir, Turkey; Department of Infectious Diseases and Clinical Bacteriology, Faculty of Medicine, Cumhuriyet University, Sivas, Turkey; Department of Clinical Microbiology, Section of Clinical Virology, Gulhane Military Medicine Academy, Ankara, Turkey; Department of Food Engineering, Faculty of Engineering, Cumhuriyet University, Sivas, Turkey; Cumhuriyet University Medical Faculty Research Center (CUTFAM), Cumhuriyet University, Sivas, Turkey; Cumhuriyet University Vocabulary School, Cumhuriyet University, Sivas, Turkey; Department of Virology, Refik Saydam Hifzissiha Institute, Turkish Ministry of Health, Ankara, Turkey; Department of Internal Medicine, Section of Haematology, Faculty of Medicine, Cumhuriyet University, Sivas, Turkey

**Keywords:** CCHF, CCHFV titer, Specific IgG, TNF-α, IL-6, IL-10, IFN-γ, DIC, Mortality

## Abstract

**Background:**

Although there have been a number of studies on the pathogenesis of Crimean-Congo hemorrhagic fever (CCHF) recently, knowledge on this topic is still insufficient. This study aims to reveal the kinetics of serum CCHF virus (CCHFV) titers, serum levels of anti-CCHFV immunoglobulin (Ig)G, tumor necrosis factor (TNF)-α, interleukin (IL)-6, IL-10, and interferon (IFN)-γ in CCHF patients.

**Methods:**

In total, 31 CCHF cases (11 fatal) were studied. Serum samples were obtained daily from all patients from the time of admission and continued for a 7-day hospitalization period for serologic (ELISA), virologic (real-time PCR), and cytokine (ELISA) analysis.

**Results:**

The mean serum CCHFV titer at admission was 5.5E + 09 copies/mL in fatal cases and 5.7E + 08 copies/mL in survivors (*p* < 0.001). Compared to survivors, both the mean serum levels of IL-6 and TNF-α at admission were found to be significantly increased in fatal cases. The serum levels of IL-6, TNF-α and serum CCHFV titer at admission were significantly and positively correlated with disseminated intravascular coagulation (DIC) scores (r = 0.626, p = 0.0002; r = 0.461, p = 0.009; and r = 0.625, p = 0.003, respectively). When the data obtained from the sequential determination of CCHFV titer and levels of anti-CCHFV IgG, IL-6, TNF-α, IL-10 and IFN-γ were grouped according to the days of illness, the initial serum CCHFV titer of a fatal patient was 5.5E + 09 (copies/mL) and it was 6.1E + 09 (copies/mL) in a survivor on the 2 day of illness. While significant alterations were observed in all cytokines during the monitoring period, IL-6 levels remained consistently higher in fatal cases and TNF-α levels increased in both in fatal and non-fatal CCHF cases.

**Conclusions:**

The increased CCHFV load and higher concentrations of IL-6 and TNF-α, the presence of DIC, and the absence of CCHFV specific immunity are strongly associated with death in CCHF.

**Electronic supplementary material:**

The online version of this article (doi:10.1186/1471-2334-14-416) contains supplementary material, which is available to authorized users.

## Background

Crimean-Congo hemorrhagic fever (CCHF) is a viral hemorrhagic fever (VHF) which is caused by an RNA virus, the CCHF virus (CCHFV), belonging to the *Nairovirus* genus of the *Bunyaviridae* family. The disease transmission occurs mainly through *Hyalomma m. marginatum* tick exposure and it progresses in severe cases resulting in fever, hemorrhage, shock, and death [1–3]. It has become an important zoonosis in Africa, Asia and Europe, particularly in Turkey [3, 4]. By the year 2012, more than 7000 confirmed CCHF cases were identified in Turkey with a mortality rate of 5% (unpublished data from the Turkish Ministry of Health). The disease typically presents itself in a significant proportion of patients with coagulation disorders, fibrinolysis, petechia, ecchymosis, and uncontrolled bleeding from the mucosa and puncture sites [2, 3, 5]. The capillary endothelium and mononuclear cells are the two main targets for CCHF [3, 6–8]. Endothelial damage either may occur directly by invasion of viruses or indirectly due to chemokines and cytokines released from activated mononuclear cells [3].

In humans and primates, expression of some inflammatory mediators such as IL-6, IL-8, IL-10, TNF-α, monocyte chemoattractant protein (MCP)-1α, and nitrous oxide (NO) are triggered during VHF. Furthermore, when human cells are exposed to VHF agents in vitro, they may produce many of these inflammatory mediators. Such inflammatory mediators play an important role in fatal cases with fulminant progression and in the development of shock [5]. Previous studies revealed that serum pro-inflammatory and anti-inflammatory cytokines including TNF-α, IL-6, IL-10, IL-12, and IFN-γ were increased in patients with CCHF and levels of some cytokines were higher in the fatal cases than those were in survivors [9–11]. Likewise, the serum viral titers have also been studied in CCHF [11–16]. The kinetics of serum viral load, cytokines and CCHFV-specific antibody levels need characterization for a thorough understanding of CCHF pathogenesis during the course of the infection. This study aims to reveal the kinetics of serum CCHFV load, anti-CCHFV IgG titers and cytokines including TNF-α, IL-6, IL-10, and IFN-γ by daily simultaneous sequential estimation in patients with CCHF and analyze their contribution to the pathogenesis of this disease.

## Methods

### Study design and patients

This prospective clinical and laboratory study has been performed in 31 adult CCHF patients (11 fatal, 20 non-fatal) who were hospitalized and followed-up in the Service of Infectious Diseases, Cumhuriyet University Hospital, Sivas, Turkey. The diagnosis of CCHF was confirmed by real-time PCR. Written informed consent was obtained from all participating CCHF patients or their relatives. This study was approved by the Human Ethics Committee of Cumhuriyet University.

### Serum collection and diagnostic tests for CCHF

Three sets of serum samples were obtained from all CCHF-suspected patients by venipuncture at admission and serum sampling with double sets was continued daily for 6 days (7 days in total). Serologic, virologic and cytokine (IL-6, IL-10, TNF-α, and IFN-γ) analysis were implemented wherever possible. One sample obtained from each patient at admission was sent to the Refik Saydam Hygiene Institute (RSHM, CCHF reference center), Ankara, Turkey for serologic (anti-CCHFV IgM, ELISA IgM capture assay) and virologic (double antibody sandwich capture assay) analysis. The reagents used for these tests were kindly provided by the Centers for Diseases Control and Prevention (CDC), USA. The remaining two serum samples from each patient were stored at -80°C until use. Further sampling of serum was based on the positive results for serologic (anti-CCHFV IgM) and/or virologic analysis, which were obtained from the RSHM within 24 – 48 hrs. Double sets of serum samples from patients were also stored at -80°C for cytokine and anti-CCHFV IgG antibody estimation.

### Assays of serum CCHFV titers, anti-CCHFV IgG antibody, and cytokine levels

At the end of the study and serum collection process, serum samples were transported on dry ice to the virology laboratory of Gulhane Military Faculty of Medicine (GATA), Ankara, Turkey for CCHFV titer estimations. A one-step real-time reverse transcriptase (RT)-PCR (7500 real time PCR system, Applied Biosystem, Foster, CA, USA) was used to monitor the viral load, as described in Yapar, et al. [12] and the results were expressed as copy/mL. The other serum samples were used to measure serum cytokine (BioSource® CA, USA) and anti-CCHFV IgG (Vector-Best Laboratories, Novosibirsk, RU) levels using ELISA test kits. Each sample was measured in duplicate and cytokine levels were expressed as picogram per milliliter. The serum levels of anti-CCHFV IgG antibodies were expressed as optical density. The sequential IgG determination could be performed only in 20 (9 fatal) patients due to death of some patients and insufficient serum samples from others. The cytokine estimation was done according to the manufacturer’s instructions at the Cumhuriyet University Medical Faculty Research Center Class 2 biological safety cabinets and strict barrier precautions (N95 mask, glow, gown etc.) were used in both centers during the course of the study.

### Diagnostic laboratory tests and patient follow-up

For each patient, complete blood count, blood aspartate aminotransferase (AST), alanine aminotransferase (ALT), lactate dehydrogenase (LDH), and creatine phosphokinase (CPK) levels were measured on a daily basis. Blood urea nitrogen (BUN), creatinine, albumin, prothrombin time (PT), activated partial thromboplastin time (aPTT), international normalized ratio (INR), fibrinogen, fibrin degradation products and D-dimer were measured every other day. Double sets of blood cultures, serum C-reactive protein (CRP) levels (nephelometric method) and erythrocyte sedimentation rate (ESR) were measured at admission, and these were repeated when needed. All patients were also evaluated for brucellosis, viral hepatitis (A, B and C) malaria, and hematological malignancy suggesting CCHF. Disseminated intravascular coagulation (DIC) score for each CCHF patient was calculated according to the recommendations of the International Society on Thrombosis and Hemostasis (ISTH) scoring system [17]. When the achieved score was ≥5, the CCHF patient was considered compatible with DIC [17]. Patients who had a history of diseases, acute infection, anti-inflammatory drug usage, pregnancy, a recent diagnosis of diabetes mellitus, viral hepatitis, brucellosis and those who had negative test results for CCHF were excluded from the study. All patients received standard medical care including fluid (crystalloid or synthetic colloid, intravenously), blood products (single random donor or apheresis platelet suspensions, fresh frozen plasma, erythrocyte suspensions), cardiovascular, and respiratory support when needed. All the patients were followed-up until discharge from the hospital or death.

### Statistical analysis

All the demographics, clinical and routine laboratory data at admission, data obtained from consecutive measurements of serum CCHF titers, cytokine levels, CCHF serology, other routine laboratory tests, and outcome data were entered into a statistics program, Statistical Package for Social Sciences (SPSS Inc. Chicago, IL, USA). The Mann-Whitney *U*-test was used for group comparisons. Categorical variables were compared using the Chi-square test, although Fisher’s exact test was used when the data was sparse. In the assessment of the relations between the variables, Pearson and Spearman correlation coefficients were calculated. Tests were two-tailed and a difference of *p* < 0.05 was accepted as statistically significant. Charts were plotted using GraphPad Prism v4.0 (GraphPad Software, San Diego, CA, USA). Data were presented as frequency and percent or mean ± standard error and (range) as appropriate.

## Results

### Patient characteristics

In total, 31 CCHF patients (11 fatal; 16 male) were studied. Close contact with livestock (87.1%), a tick-bite history (64.5%), and living in the CCHF epidemic area were the risk factors for having acquired the infection. The mean age was 53.8 ± 4.5 (25-82) years in the fatal CCHF group, and 45.9 ± 3.6 (19-81) years in the non-fatal CCHF group (*p* > 0.05). The time to hospitalization after the onset of symptoms was significantly longer in the non-fatal group than that of fatal group (5.9 ± 0.4 *vs.* 4 ± 0.4 days; *p* = 0.011). During the follow-up period, 2 patients died on day 1 of hospitalization, 2 patients died on day 2, 1 patient each died on days 4, 5, 6, 8, and 9.

### Clinical and laboratory findings

Comparison of symptoms and signs between fatal and non-fatal CCHF patient groups at admission is presented in Table 1. The blood hemoglobin (12.4 *vs.* 13.8 g/dl), albumin (2.8 *vs.* 2.9 g/dL) levels and ESR (27 *vs.* 21 mm/hr) were identical between fatal and non-fatal groups *(p >* 0.05 for all comparisons). Routine tests of blood coagulation, such as PT and aPTT were revealed coagulation to be significantly prolonged and the INR value was significantly higher in the fatal group when compared to that of the non-fatal group (PT: 21.7 *vs.* 14 s, *p* < 0.001; aPTT: 49.2 *vs.* 34.2 s, *p* = 0.001; INR: 1.9 *vs.* 0.97, *p* < 0.001). Furthermore, according to the recommendation of ISTH DIC scoring system [17], all (100%) fatal, but 5 (25%) survivors had DIC (*p* < 0.0001) with the DIC score in the fatal group being significantly higher than that in the survivors (6.8 *vs.* 3.2, *p* < 0.0001). The blood CRP and bilirubin levels were also found to be significantly higher in the fatal CCHF patients than that in the survivors (CRP: 46 *vs.* 6.3 mg/L, *p* < 0.001; bilirubin: 1.35 *vs.* 0.84 mg/dL, *p* = 0.034).

**Table 1:** **Comparison of the symptoms and signs between the non-fatal and fatal CCHF patients at admission**

Symptoms and signs, ***n***(%)	All patients	Non-fatal	Fatal	P-value
	(n = 31)	(n = 20)	(n = 11)	
Weakness	31 (100)	20 (100)	11 (100)	1.0
Myalgia	29 (93.5)	19 (95)	10 (90)	1.0
Fever	29 (93.5)	18 (90)	11 (100)	1.0
Nausea and/or vomiting	24 (77.4)	14 (70)	10 (90)	0.37
Chills	23 (74.1)	19 (95)	4 (36.4)	0.0009
Headache	22 (70.9)	12 (83)	10 (90)	0.11
Diarrhea	16 (51.6)	13 (65)	3 (27.2)	0.07
Rash	16 (51.6)	9 (45)	7 (63.9)	0.46
Bleeding	13 (41.9)	7 (35)	6 (54.5)	0.45
Facial hyperemia	10 (32.2)	7 (35)	3 (27.2)	1.0
Petechiae or ecchymosis	8 (25.5)	4 (20)	4 (36.3)	0.41
Hepatomegaly	4 (12.9)	3 (15)	1 (9.1)	1.0
Jaundice	4 (12.9)	0 (0)	4 (36.3)	0.01
Stupor	1 (3.2)	0 (0)	1 (9.1)	0.003

### Serum CCHFV titers and cytokine levels

The CCHFV titers at admission in fatal patients was found to be significantly higher than that in the non-fatal patients (5.5E + 09 ± 1.4E + 09 *vs* 5.7E + 08 ± 3.8E + 08 copies/mL, *p* < 0.0001). Fatal CCHF cases showed significantly higher IL-6 levels than did the patients in the non-fatal group (326.1 ± 74.9 *vs* 70.7 ± 17.4 pg/mL; *p* < 0.001). The serum level of IL-10 was 46.5 ± 9.6 (pg/mL) in the survivors and it was 30.5 ± 12.8 (pg/mL) in the fatal group (*p* > 0.05). The serum level of TNF-α was found to be 161.3 ± 26.3 (pg/mL) in the fatal group and 77.3 ± 7.2 (pg/mL) in the survivors (*p* < 0.001). Serum levels of IFN-γ were comparable between the two groups (fatal *vs* survivor, 18.6 ± 8.1 *vs* 9.9 ± 2.2 pg/mL; *p* > 0.05).

### Correlation analysis among the cytokines, CCHFV titers and routine laboratory tests at admission

We found significant positive correlations between serum IL-6 and blood CRP levels, PT, aPTT, INR values, DIC scores, and serum TNF-α levels of all CCHF patients (r = 0.489, *p* = 0.005; r = 0.462, *p* = 0.009; r = 0.692, *p* < 0.0001; r = 0.544, *p* = 0.0015; r = 0.626, *p* = 0.0002; and r = 0.356, *p* = 0.04, respectively) (Additional file 1: Figure S1(A-F). In addition, there was also a positive correlation between serum IL-6 and AST (r = 0.575, *p* = 0.0007) levels (Additional file 1: Figure S1G). While no significant correlation between serum TNF-α and blood PT, aPTT and INR values existed, a positive correlation between serum TNF-α levels and DIC scores was observed (r = 0.461, *p* = 0.009) (data not shown). Further, there was no significant correlation between the serum levels of cytokines studied and the blood platelet counts. The serum CCHFV titer was significantly and positively correlated with serum IL-6, TNF-α and blood CRP levels, PT, aPTT, INR values and DIC scores (r = 0.809, *p* < 0.0001; r = 0.510, *p* = 0.0047; r = 0.540, *p* = 0.0027; r = 0.657, *p* = 0.0001; r = 0.486, *p* = 0.0075; r = 0.739, *p* < 0.0001 and r = 0.625, *p* = 0.0003, respectively) (Additional file 2: Figure S2A-G).

### Kinetics of serum CCHFV titers, anti-CCHFV IgG levels, selected routine laboratory tests and serum cytokine concentrations

**Figure 1:**
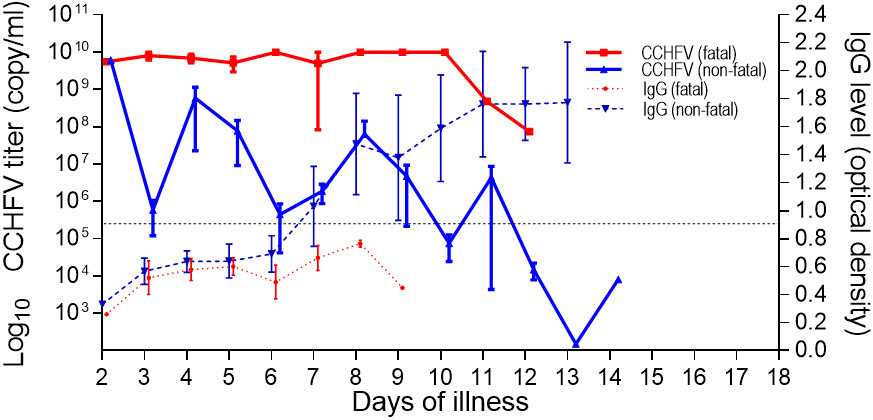
**Kinetics of serum virus titers (left y-axis) in logarithmic scale and CCHFV specific IgG antibodies (right y-axis) by days of illness in fatal and non-fatal CCHF groups.** Data are expressed as mean ± SE. The threshold optic density value for anti-CCHFV IgG as indicated by the manufacturer is indicated by a dotted line.

**Figure 2:**
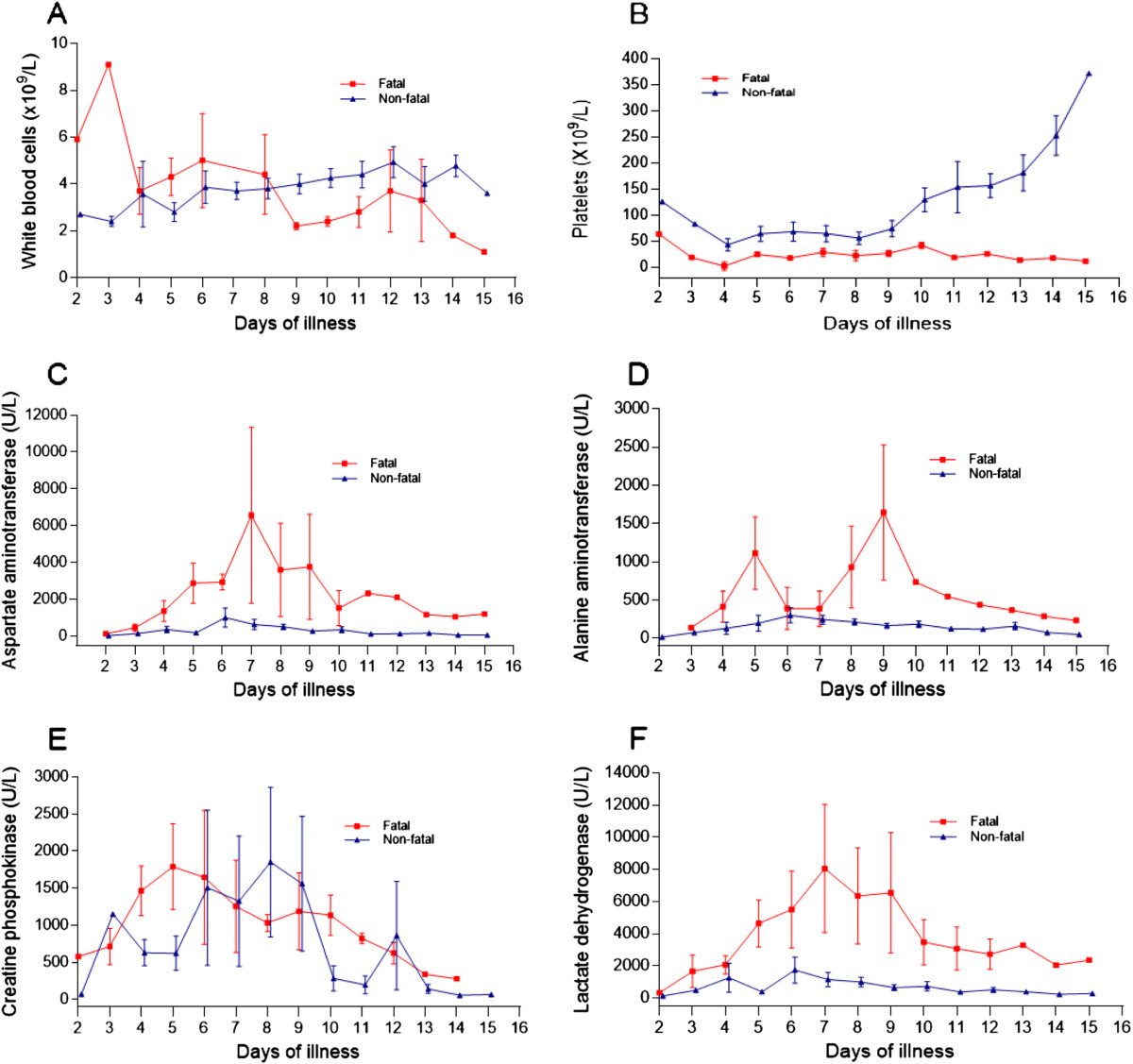
**Kinetics of some routine laboratory analysis on different days of illness in fatal and non-fatal CCHF cases**. **(A)** White blood cells; **(B)** Platelets; **(C)** Aspartate aminotransferase; **(D)** Alanine aminotransferase; **(E)** Creatine phosphokinase; and **(F)** Lactate dehydrogenase. Data are expressed as mean ± SE.

**Figure 3:**
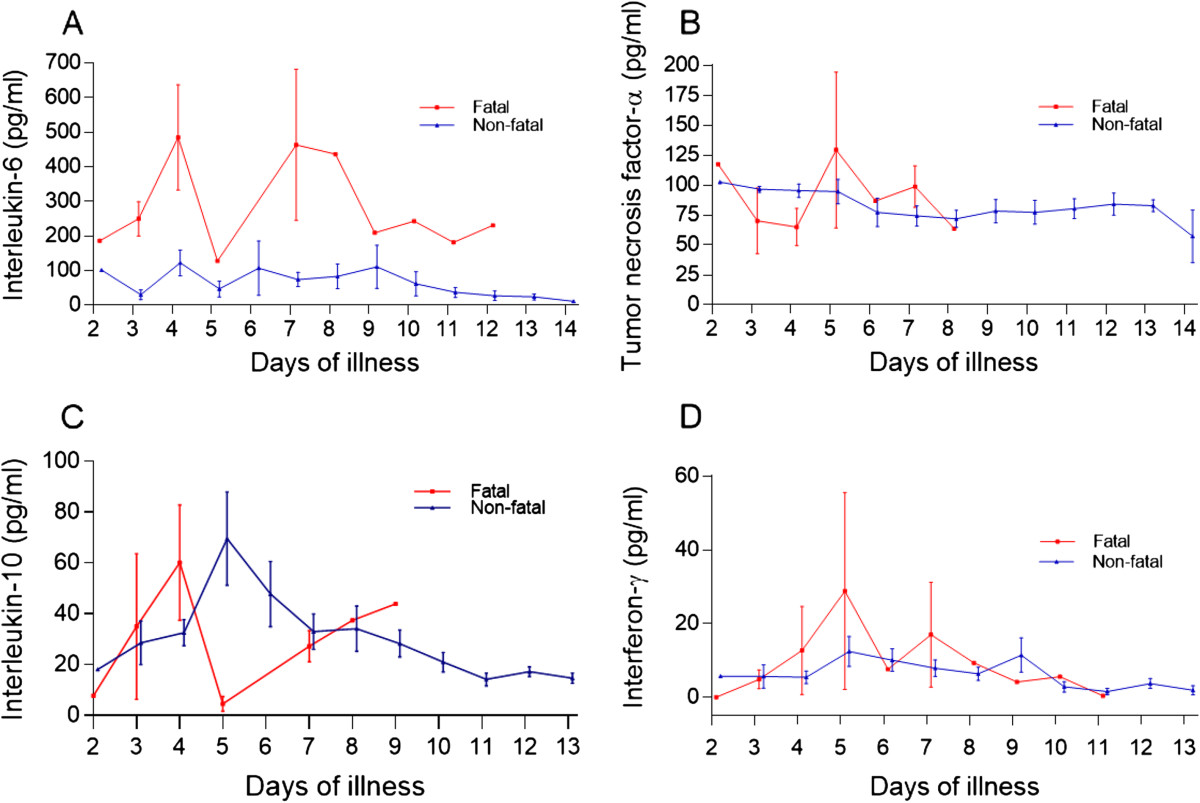
**Kinetics of serum cytokine concentrations over multiple days of illness in fatal and non-fatal CCHF cases.** **(A)** Interleukin-6; **(B)** Tumor necrosis factor-alpha; **(C)** Interleukin-10; and **(D)** Interferon-gamma. Data are expressed as mean ± SE.

Data obtained from the analysis of serum samples in the fatal and the survivor groups were pooled according to the days of illness and the kinetic changes of serum CCHFV titers and anti-CCHFV IgG levels (Figure 1), selected routine laboratory tests (Figure 2), and serum concentrations of the cytokines (IL-6, TNF-α, IL-10 and IFN-γ), studied (Figure 3) were plotted. The day of onset of symptoms including fever and/or chills was assumed to be the first day of illness for each patient.

It was observed that the earliest day of admission among the patients was the second day of illness (one patient each in the fatal and in the survivor group). The initial viral titer in the serum of fatal and non-fatal patients was 5.5E + 09 and 6.1E + 09 copies/mL, respectively. While CCHFV titers appeared to decline over time, no clear trend could be recognized in the fatal patients. A great variation was observed on day 5 of illness; however, the viral titers remained high, and by day 12 only a 2-log decline was observed from the baseline in this group of patients. A female CCHF patient, who died on day 12 of the illness, had a viral titer of 7.4E + 07 copies/mL on the same day. The viral titers also appeared to decline over time in the survivors. However, in contrast to fatal patients, a clear declining trend was observed for serum viral titers in this group of patients. A male survivor had a viral titer of 8.1E + 03 copies/mL on day 14 of the illness. An approximate 6-log decline of viral titers from the baseline was observed in the survivors. In contrast to the fatal patients, the serum anti-CCHFV IgG levels of survivors reached the optic density level of 0.90 (the threshold level by the manufacturer) on day 8 of the illness and increased rapidly during the monitoring period. The analysis of two serum samples obtained from the days 8 and 9 of the illness of a fatal patient who died on day 15 of illness (9 days after hospitalization) showed insufficient IgG levels (Figure 1).When the kinetics of WBC, blood platelet count, serum AST, ALT, CPK and LDH levels were analyzed (Figure 2A-F), it was seen that while the blood platelet count of the survivors reached the normal range on day 11 of the illness, the fatal patients remained thrombocytopenic until death. Although great variations were observed on certain days in AST, ALT, and LDH levels in the fatal patients during the monitoring period, both liver enzymes and LDH were found to be higher in the fatal group than in the survivors. Two apparent peaks in the ALT level were observed on days 5 and 9 of CCHF in the fatal group. (Figure 2D).The serum levels of IL-6 in both fatal and non-fatal groups were found to be increased on day 2 and continued to increase up to day 4 after the onset of fever in the fatal group. Great variations were observed on day 4 and 7 and the cytokine level remained highest in this group of patients. Although variations were determined on different illness days, the IL-6 levels gradually decreased during the convalescence phase and the lowest level was determined on day 14 in the non-fatal group (Figure 3A). Interestingly, the serum TNF-α concentration for both groups of patients was increased in the acute phase and remained as high by day 14 in the non-fatal group. A peak concentration with a great variation for this cytokine was determined on day 5 of CCHF in fatal patients (Figure 3B). The level of IL-10 gradually increased in both groups and peak serum levels were determined on day 4 and 5 in both groups, respectively. In contrast to the non-fatal group, a low level of IL-10 was determined on day 5 in the fatal group (Figure 3C). The IFN-γ level in both groups rapidly increased from day 2 to day 5 and then decreased up to day 11 in the fatal group and day 13 in the non-fatal group respectively (Figure 3D).

## Discussion

Although the number of studies on CCHF pathogenesis increased significantly, data in this field are still insufficient. Therefore, in this study, some of the routine laboratory tests, serum cytokines, CCHFV titers and virus specific IgG antibodies along with their kinetics were investigated. We observed that some of the routine test parameters were significantly altered compared to those of the survivors. We also observed that certain parameters of the survivors progressively improved especially during the second week of the disease. Our findings are in agreement with other reports where both leukopenia and thrombocytopenia are frequently detected in the CCHF patients [2, 3, 10, 18]. By contrast to the survivors, prolonged coagulation tests and higher INR values –both of which were reported to be poor indicators for CCHF by a previous study [4]- were more common in the fatal cases. In the fatal CCHF cases, the serum levels of ALT had two peaks on days 5 and 9 of the disease. We think that these peaks may have occurred during the inflammatory response and subsequent hepatocyte damage, and by direct damage to the hepatocytes by CCHFV. The positive correlation between IL-6 and the AST concentration in this study also supports this suggestion. The virus has both hepatotropic and neurotropic characteristics and it has been detected in both liver and brain tissues in a mouse model [19] and within the hepatocytes from fatal CCHF cases [7]. Furthermore, the source of the prolonged viremia in a mouse model was found to be the liver [19]. While fatal patients had thrombocytopenia, the blood platelet counts of the survivors started to increase on day 9 and reached the normal range on day 11 of the illness. We think that development of specific IgG antibodies against CCHFV in the host results in the symptoms of the disease subsiding and normalizations of laboratory test values in the survivors, as shown previously by a clinical study [20].

Except for IL-10 and IFN-γ, the serum TNF-α and IL-6 levels were found to be significantly higher for the fatal cases than were those of the survivors at admission. In addition, the absence of prominent reductions in the sequential determinations of TNF-α in both groups and IL-6 in fatal group strongly suggests the existence of a profound inflammatory response in the fatal CCHF cases and this was evident until death. The results of this study regarding the cytokine levels in CCHF at admission are in agreement with previously reported studies performed on humans [9–11]. It is believed that CCHFV changes the homeostatic mechanisms in the host through two different pathways. One of them is a direct effect on the endothelial cells associated with homeostasis and platelets and the other one is an indirect effect on the endothelium via immunological and inflammatory pathways [3, 21]. In a previous immunohistochemical study, the virus has been shown in endothelial cells in patients with CCHF [7]. It is likely that the functional impairment of the endothelial cells depends on the circulating mediators which appear during the acute phase of the disease. It has previously been shown that cells infected with filoviruses release inflammatory mediators including TNF-α that may disrupt endothelial permeability and integrity [22]. Subendothelial edema observed in the CCHF patients is probably associated with TNF-α [23]. Furthermore, TNF-α has anti-fibrinolytic effects by inhibiting the physiological anticoagulation pathways, and the production of plasminogen activators and the plasminogen activator inhibitor (PAI)-I are induced by this cytokine. These effects facilitate DIC development in the host. Another important pro- and anti-inflammatory cytokine, IL-6, is the actual mediator for the activation of the coagulation system in the host [24, 25]. Furthermore, tissue factor (TF)-mediated thrombin formation which is initiated by the IL-6 and TF/activated factor VII complex, has an important role in the development of DIC, similar to TNF-α [9, 26, 27]. In this study 16 (51.6%) out of the 31 patients (11 fatal, 5 survivors) had DIC at admission and nearly 69% of the 16 patients with DIC died during the follow-up period. We also observed that the presence of DIC is an indicator for poor prognosis in patients with CCHF, as previously described [18, 28].

IL-10, a potent anti-inflammatory mediator of vascular damage, controls coagulation by inhibiting TF expression on the monocyte surface [29]. It also plays a role in the efficiency of the humoral immune response by amplification of Ig secretion by activated B-lymphocytes and in B-cell differentiation [30]. The exact cause of thrombocytopenia in VHF has not been demonstrated yet. However, the level of IL-10 has been found to be significantly correlated with thrombocytopenia in Ebola virus infection [31]. Moreover, a fatal CCHF case that had a high level of IL-10 and severe thrombocytopenia has been defined [9]. In contrast to the results of this study, fatal Ebola patients had higher serum IL-10 levels than the survivors [31]. IFN-γ, which is secreted by activated T-cells and natural killer (NK) cells, is responsible for the inflammatory response and viral immunity via macrophage activation [32]. In contrast to our findings, a previous study showed significantly higher levels of this cytokine in CCHF [11].

Recent studies indicate that CCHFV titer in serum samples is important to predict the clinical severity and the fatal outcome in CCHF [11, 13, 14]. A clinical study from Kosovo suggests a viral load of >10^8^ copies/mL as a strong factor for differentiating CCHF patients who died from those who survived [13]. Another report from Turkey showed that viral load ≥10^9^ copies/mL could be considered to predict a fatal outcome with 88.9% sensitivity and 92.6% specificity [14]. Other studies also revealed that the fatal CCHF cases had a higher blood viral load [15, 16]. In our study, we determined that the admission serum CCHFV titer in both groups was found to be higher than the ranges mentioned above. In this study, we also determined that 8 (72.2/%) out of 11 fatal and 3 (15%) out of 20 non-fatal CCHF cases had the virus titer of >10^8^ copies/mL at admission (data not shown). Furthermore, according to kinetics of serum CCHFV titers, all but one, of the fatal cases had a virus titer of >10^8^ copies/mL until death. The observation of only a 2-log decline in the CCHFV titer from the baseline in the fatal group might have occurred due to inappropriate immune response both in the early and late phase of the disease in these cases. Innate immunity is the first line of defense against viruses before adaptive immunity develops and it is characterized by the production of type I IFN, which is crucial for limiting the early replication and spread of viruses. CCHFV is one of the viruses sensitive to type I IFN, and it delays the IFN response [33]. Furthermore, CCHFV challenge of type I IFN receptor-knockout mice results in a fatal outcome and higher CCHFV titers [34, 35]. This study showed that all survivors develop a sufficient amount of specific IgG antibody after 8 days of fever onset. Due to the limitation in serum sample amounts, we could not estimate IgG antibody levels in the fatal patients after day 9 of the illness. However, when compared to survivors, the consistently higher CCHFV titer in a fatal CCHF patient after day 8 of the illness strongly suggests insufficient amount of specific IgG antibody against the virus. The initial virus specific antibody activity can be assessed by ELISA IgG and IgM after 2 days of the onset of CCHF symptoms and the levels increase between days 7 to 9 of the illness in survivors [20]. Likewise Duh, et al. [13] showed that all 9 fatal and most of the severe CCHF cases had no IgG antibodies as determined by ELISA even on day 9 of the illness. Other reports also suggest insufficient IgG antibody responses in severe and fatal CCHF cases [15, 20, 23]. The lack of specific IgG antibodies in fatal cases has entailed using human CCHF hyperimmunoglobulin with a promising effect [16]. Finding a meaningful correlation between serum CCHFV titer and DIC score can be expected because the results of this study, as well as earlier ones, suggest a close relationship between CCHFV titer, and both clinical severity and fatal outcome in CCHF [11, 13–16].

## Conclusion

In conclusion, the results of this study showed that higher CCHFV titers and overproduction of TNF-α and IL-6, (a phenomenon called cytokine storm), the presence of DIC, and the absence of CCHFV specific immunity have important roles in the pathogenesis of CCHF and are strongly associated with a fatal outcome. In addition, the levels of serum IL-6, TNF-α, and CCHFV titers correlated with the existence of DIC, subsequently leading to hemorrhages and death. Lower serum CCHFV titers in the survivors than in the fatal patients during the course of CCHF may suggest that the disease is being controlled both in the acute and convalescent phases by the innate and adaptive immune systems. The difference between innate and adaptive immune responses of the survivor and fatal CCHF patients need to be elucidated to understand thoroughly the pathogenesis of CCHF.

## Electronic supplementary material

Additional file 1: Correlation between serum interleukin-6 level (pg/mL) at the time of admission and (A) C-reactive protein, (B) Prothrombin time (C), Activated partial thromboplastin time, (D) International normalized ratio, (E) Disseminated intravascular coagulation score, (F) Tumor necrosis factor-alpha, and (G) Aspartate aminotransferase.(DOC 2 MB)

Additional file 2: Correlation between serum virus titer (copy/mL) at the time of admission and (A) C-reactive protein, (B) Prothrombin time (C), Activated partial thromboplastin time, (D) International normalized ratio, (E) Disseminated intravascular coagulation score, (F) Serum tumor necrosis factor-alpha, and (G) serum interleukin-6.(DOC 2 MB)

Below are the links to the authors’ original submitted files for images.

Authors’ original file for figure 1

Authors’ original file for figure 2

Authors’ original file for figure 3

Authors’ original file for figure 4

Authors’ original file for figure 5
