# Supplementary figures and images for: Sequential determination of serum viral titers, virus-specific IgG antibodies, and TNF-α, IL-6, IL-10, and IFN-γ levels in patients with Crimean-Congo hemorrhagic fever
Source: BMC Infect Dis. 2014 Jul 28;14:416. doi: 10.1186/1471-2334-14-416 (PMC4133611; doi:10.1186/1471-2334-14-416)

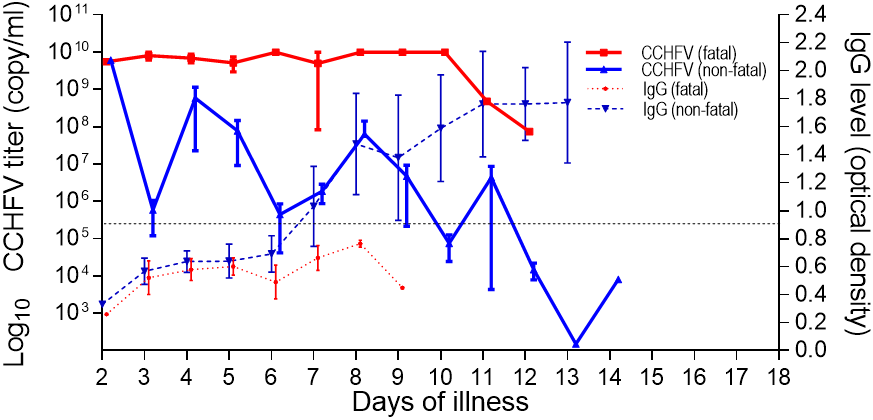

Supplement: Supplementary file 3 — Authors’ original file for figure 1 [file 12879_2014_3737_MOESM3_ESM.tif]

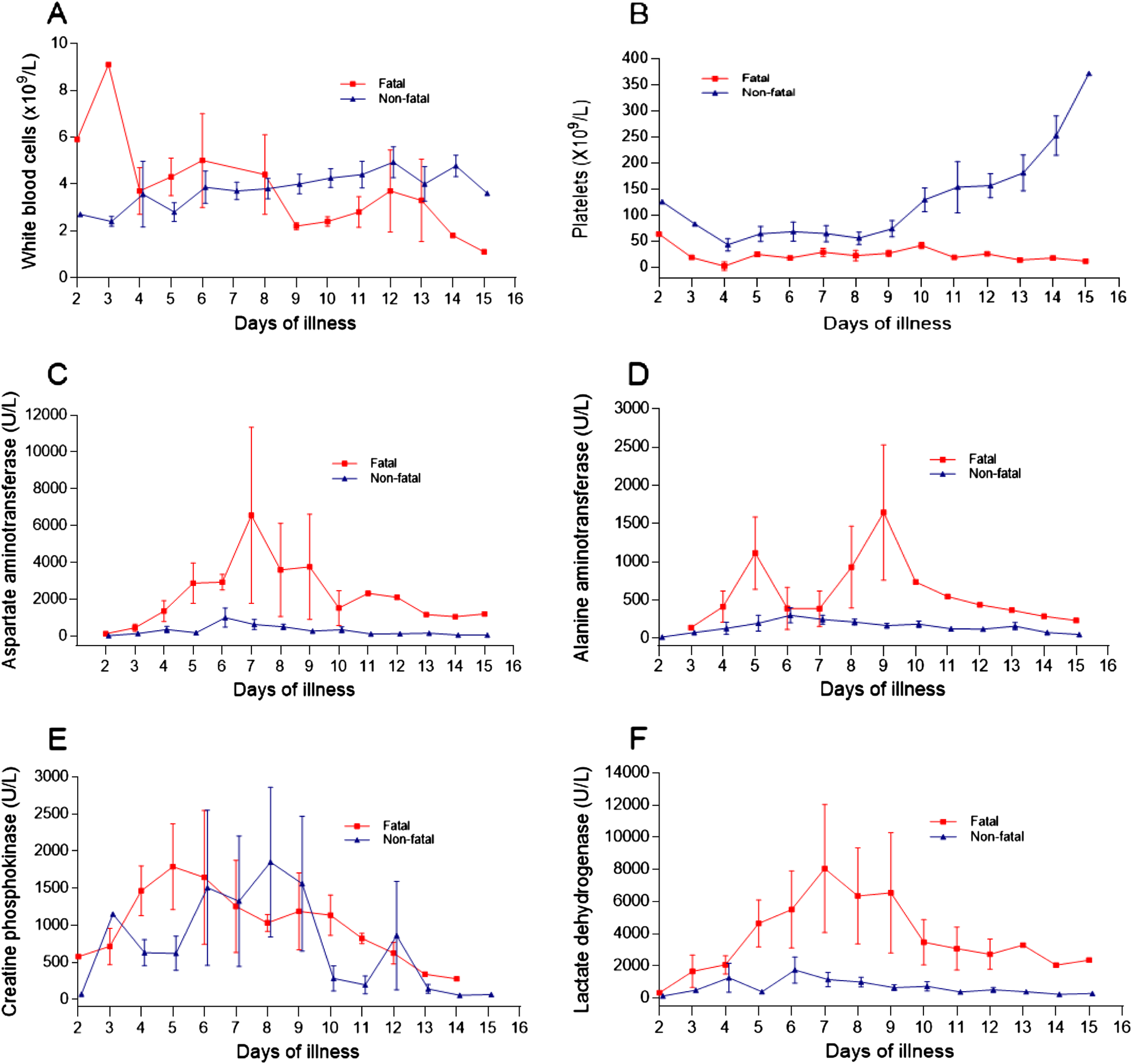

Supplement: Supplementary file 4 — Authors’ original file for figure 2 [file 12879_2014_3737_MOESM4_ESM.tiff]

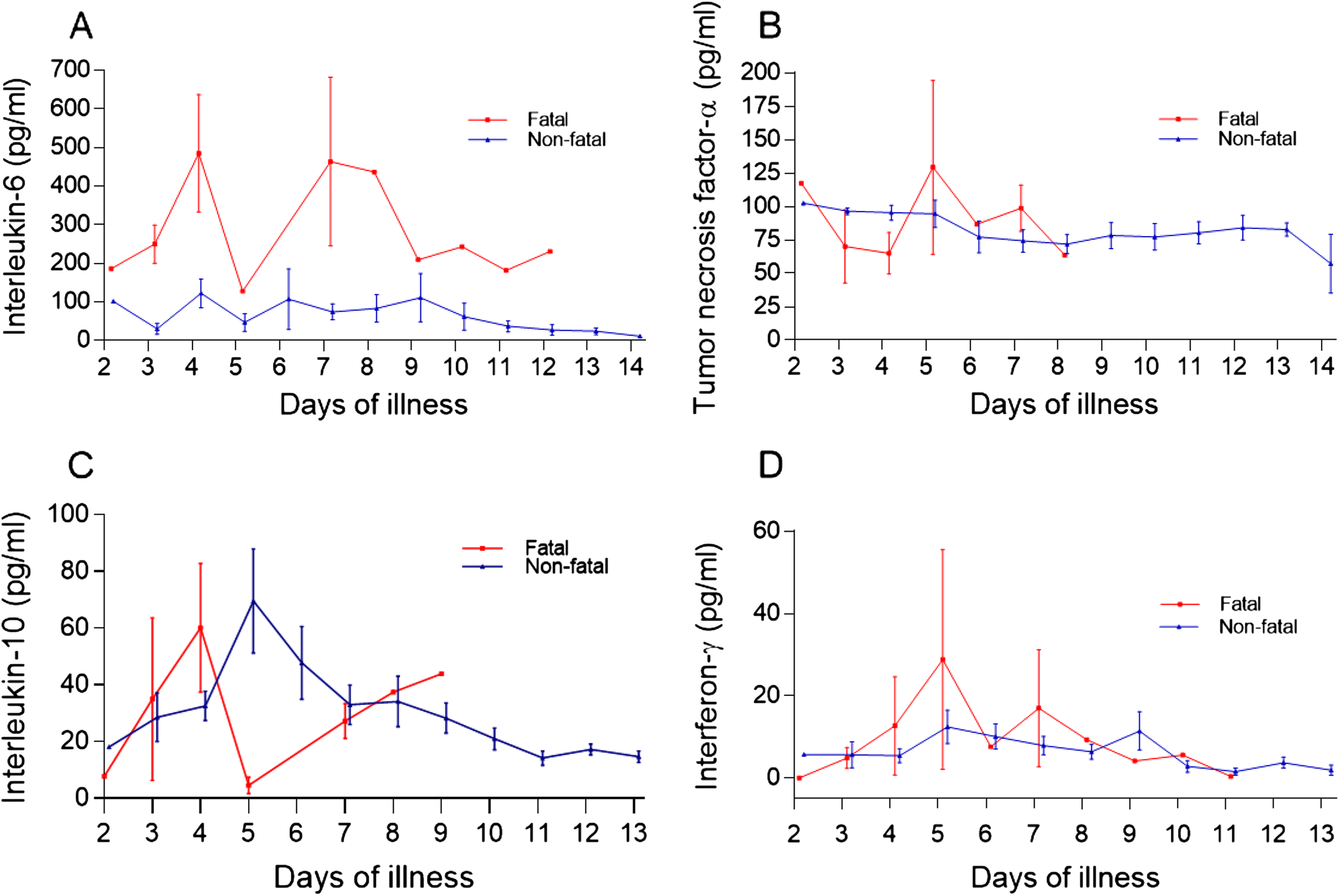

Supplement: Supplementary file 5 — Authors’ original file for figure 3 [file 12879_2014_3737_MOESM5_ESM.tiff]
