# Supplementary material for: Sequential determination of serum viral titers, virus-specific IgG antibodies, and TNF-α, IL-6, IL-10, and IFN-γ levels in patients with Crimean-Congo hemorrhagic fever
Source: BMC Infect Dis. 2014 Jul 28;14:416. doi: 10.1186/1471-2334-14-416 (PMC4133611; doi:10.1186/1471-2334-14-416)
Supplement: Supplementary file 2 [file 12879_2014_3737_MOESM1_ESM.doc]

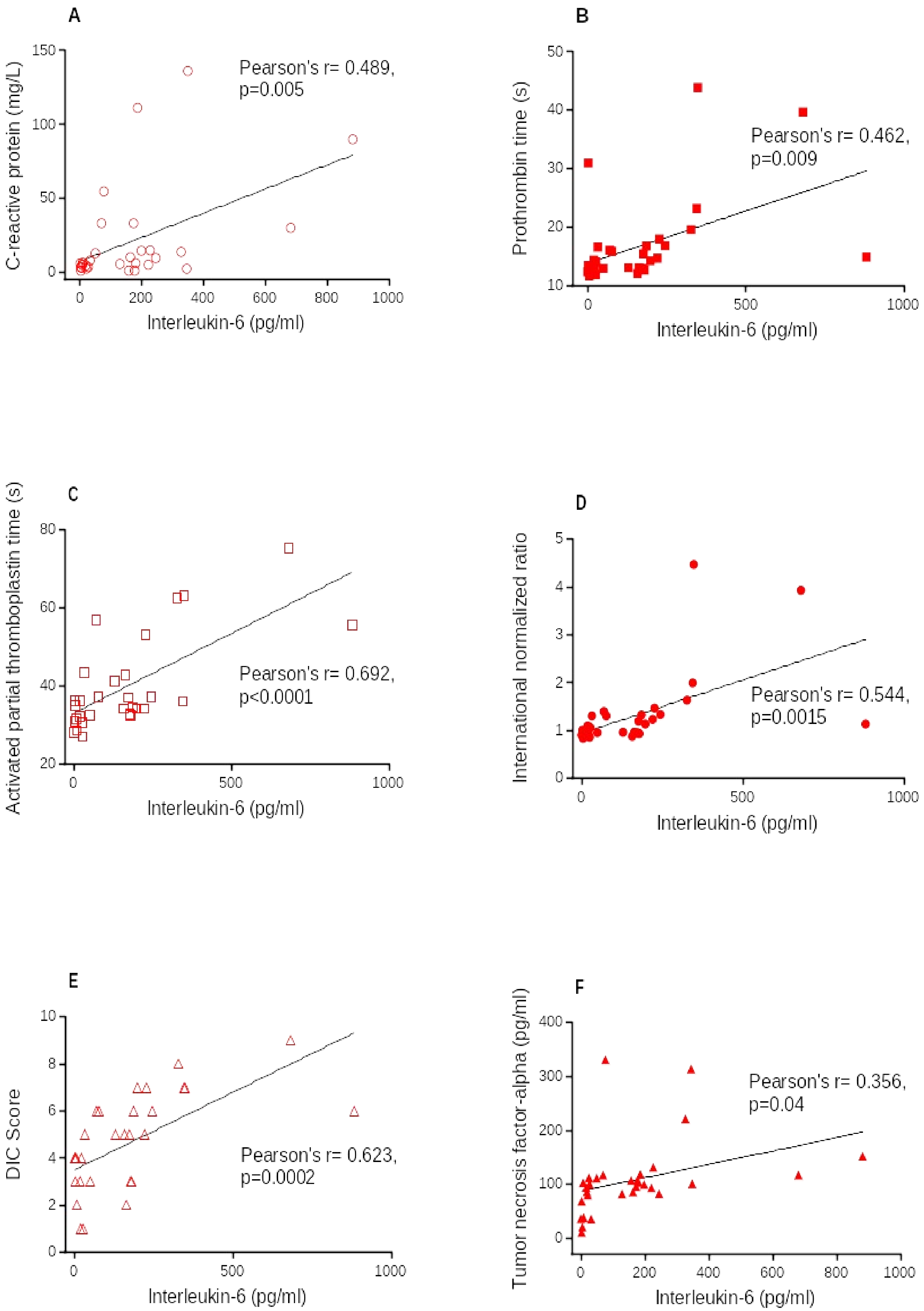
A
C-reactive protein (mg/L)
0
50
100
150
0
200
400
600
800
1000
Interleukin-6 (pg/ml)
Pearson's r= 0.489,
p=0.005
B
Prothrombin time (s)
10
20
30
40
50
0
500
1000
Interleukin-6 (pg/ml)
Pearson's r= 0.462,
p=0.009
C
Activated partial thromboplastin time (s)
20
40
60
80
0
500
1000
Interleukin-6 (pg/ml)
Pearson's r= 0.692,
p<0.0001
D
International normalized ratio
0
1
2
3
4
5
0
500
1000
Interleukin-6 (pg/ml)
Pearson's r= 0.544,
p=0.0015
E
DIC Score
0
2
4
6
8
10
0
500
1000
Interleukin-6 (pg/ml)
Pearson's r= 0.623,
p=0.0002
F
Tumor necrosis factor-alpha (pg/ml)
0
100
200
300
400
0
200
400
600
800
1000
Interleukin-6 (pg/ml)
Pearson's r= 0.356,
p=0.04

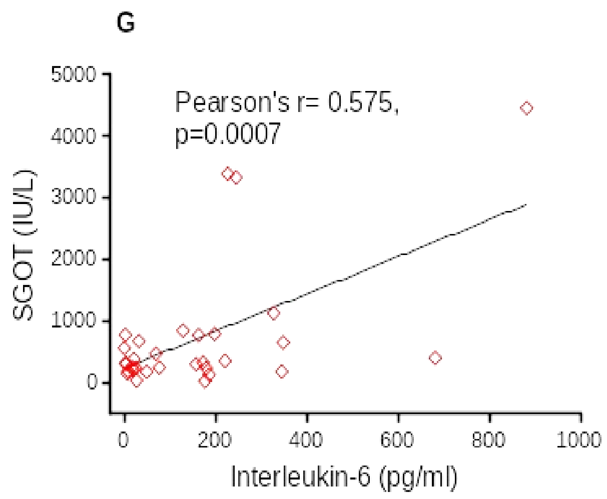
G
Pearson's r= 0.575,
p=0.0007
SGOT (IU/L)
5000
4000
3000
2000
1000
0
0
200
400
600
800
1000
Interleukin-6 (pg/ml)
