# Supplementary material for: Sequential determination of serum viral titers, virus-specific IgG antibodies, and TNF-α, IL-6, IL-10, and IFN-γ levels in patients with Crimean-Congo hemorrhagic fever
Source: BMC Infect Dis. 2014 Jul 28;14:416. doi: 10.1186/1471-2334-14-416 (PMC4133611; doi:10.1186/1471-2334-14-416)
Supplement: Supplementary file 3 [file 12879_2014_3737_MOESM2_ESM.doc]

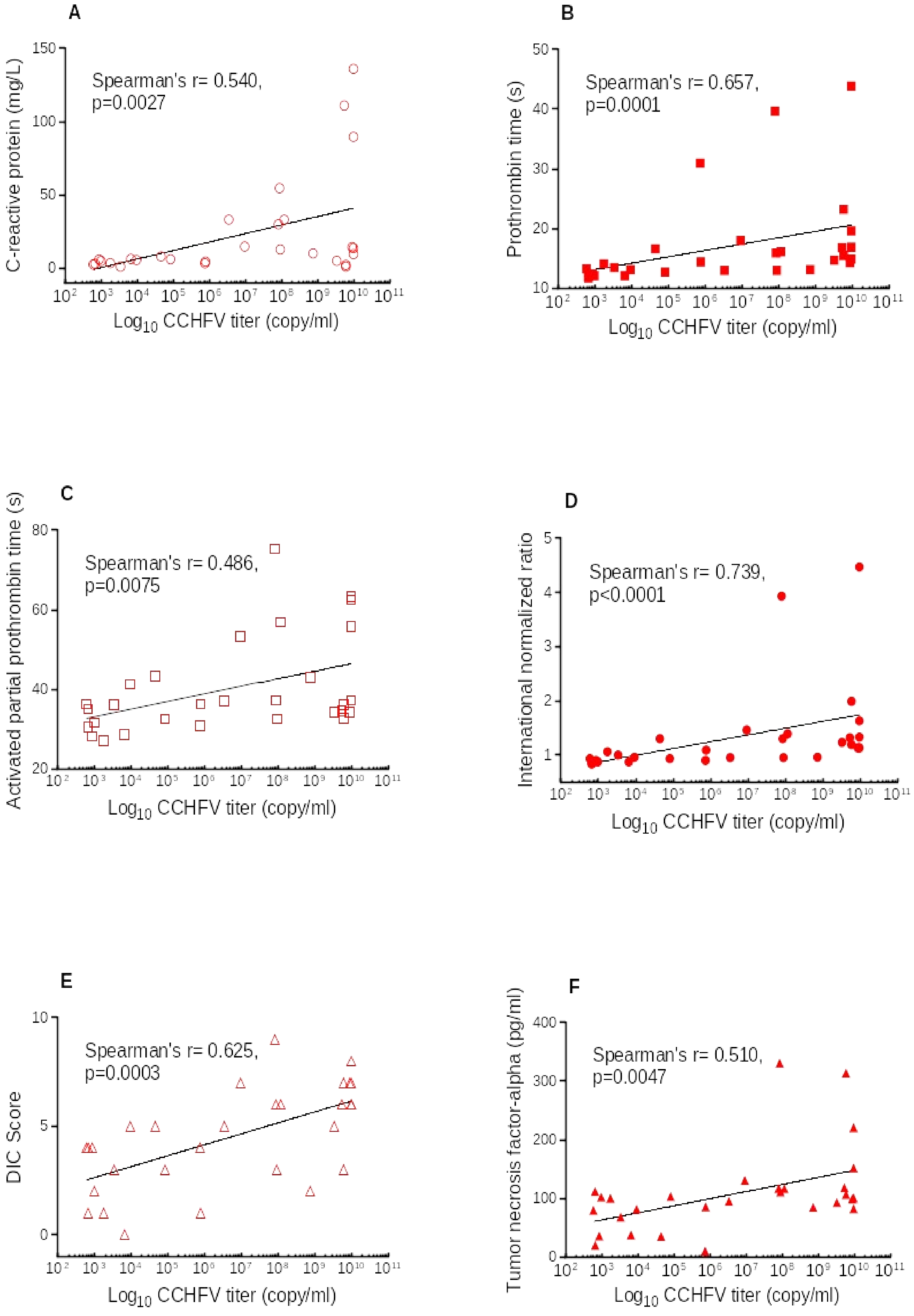

A
C-reactive protein (mg/L)
Spearman's r= 0.540,
p=0.0027
Log10 CCHFV titer (copy/ml)
B
Prothrombin time (s)
Spearman's r= 0.657,
p=0.0001
Log10 CCHFV titer (copy/ml)
C
Activated partial prothrombin time (s)
Spearman's r= 0.486,
p=0.0075
Log10 CCHFV titer (copy/ml)
D
International normalized ratio
Spearman's r= 0.739,
p<0.0001
Log10 CCHFV titer (copy/ml)
E
DIC Score
Spearman's r= 0.625,
p=0.0003
Log10 CCHFV titer (copy/ml)
F
Tumor necrosis factor-alpha (pg/ml)
Spearman's r= 0.510,
p=0.0047
Log10 CCHFV titer (copy/ml)

G

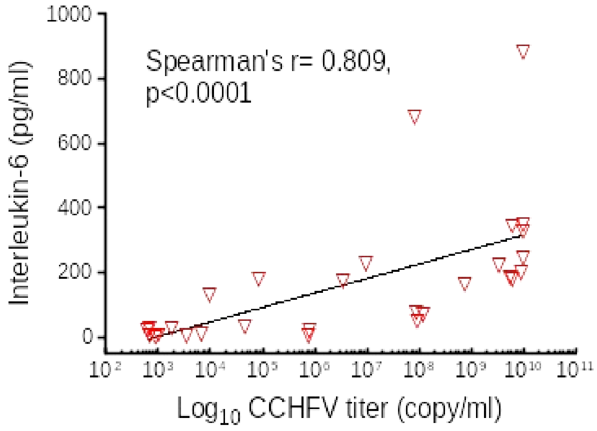
Spearman's r= 0.809,
p<0.0001
Interleukin-6 (pg/ml)
1000
800
600
400
200
0
10 2
10 3
10 4
10 5
10 6
10 7
10 8
10 9
10 10
10 11
Log10 CCHFV titer (copy/ml)
